# Production of Gluconic Acid and Its Derivatives by Microbial Fermentation: Process Improvement Based on Integrated Routes

**DOI:** 10.3389/fbioe.2022.864787

**Published:** 2022-05-16

**Authors:** Yan Ma, Bing Li, Xinyue Zhang, Chao Wang, Wei Chen

**Affiliations:** ^1^ School of Marine Science and Engineering, Qingdao Agricultural University, Qingdao, China; ^2^ Dongcheng District Center for Disease Control and Prevention, Beijing, China

**Keywords:** gluconic acid, microbial fermentation, synthetic pathway, regulatory mechanisms, agro-industrial byproducts, integrated routes

## Abstract

Gluconic acid (GA) and its derivatives, as multifunctional biological chassis compounds, have been widely used in the food, medicine, textile, beverage and construction industries. For the past few decades, the favored production means of GA and its derivatives are microbial fermentation using various carbon sources containing glucose hydrolysates due to high-yield GA production and mature fermentation processes. Advancements in improving fermentation process are thriving which enable more efficient and economical industrial fermentation to produce GA and its derivatives, such as the replacement of carbon sources with agro-industrial byproducts and integrated routes involving genetically modified strains, cascade hydrolysis or micro- and nanofiltration in a membrane unit. These efforts pave the way for cheaper industrial fermentation process of GA and its derivatives, which would expand the application and widen the market of them. This review summarizes the recent advances, points out the existing challenges and provides an outlook on future development regarding the production of GA and its derivatives by microbial fermentation, aiming to promote the combination of innovative production of GA and its derivatives with industrial fermentation in practice.

## 1 Introduction

**FIGURE 1:**
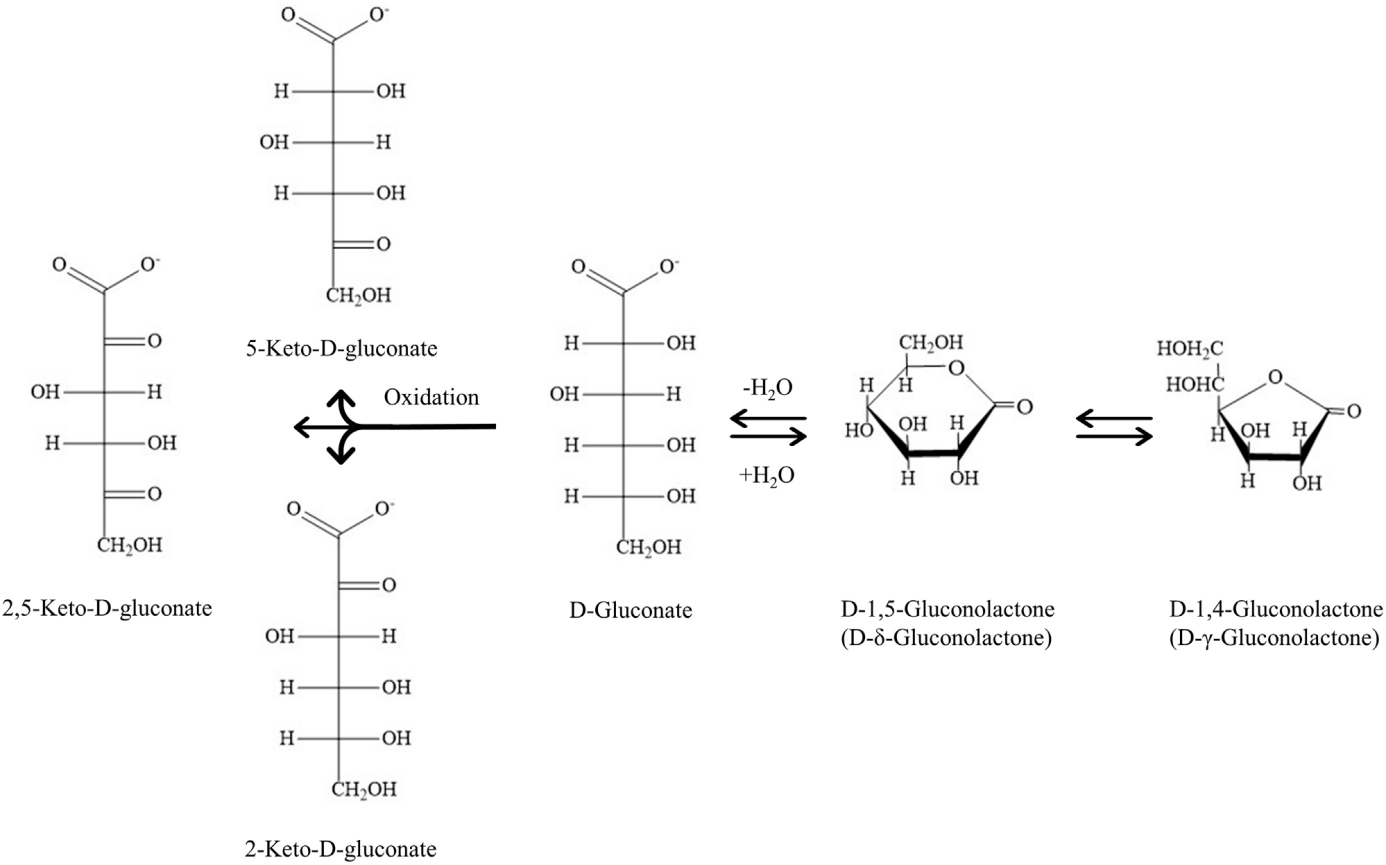
Gluconic acid (GA) and its derivatives. Chemical equilibrium of GA and its lactones and GA transformation to keto-gluconates by a strong oxidant in aqueous solution.

Gluconic acid (GA, C_6_H_12_O_7_, 2,3,4,5,6-pentahydroxyhexanoic acid), a bio-based additive, has been widely used in the food, medicine, textile and construction industries. GA is an aldonic acid derived from *ß*-d-glucose via a site-specific oxidation, in which the aldehyde group (-CHO) at C-1 is oxidized electrochemically or catalytically into a carboxyl group (-COOH). It is readily soluble in water with p*K*_*a*_ of 3.70 at 25°C. GA can convert into the forms of its gluconolactones (D-1,5-gluconolactone and D-1,4-gluconolactone) and gluconates in an aqueous solution where they are in equilibrium with each other (Figure 1). For example, the commercial GA aqueous solution (50%) contains about 5% of its lactones at room temperature (Lim and Dolzhenko, 2021). Under certain conditions, GA is prone to be oxidized to keto-d-gluconic acid (Hustede et al., 2012). Currently, the annual worldwide market of GA is between US$ 50 million and US$ 80 million, and its industrial consumption will probably exceed 1.2 × 10^5^ tonnes by 2024 (Ahuja and Singh, 2018). In addition, GA and its derivatives are designated as “generally recognized as safe” (GRAS) by the U.S. Food and Drug Administration (US FDA), the European Parliament and the Council Directive No. 95/2/EC and World Health Organization (WHO) (Pal et al., 2016).

Over the past 50 years, GA and its derivatives have been applied globally with different shares in the construction (45%), food (35%), medicine (10%) and other (10%) industries (Kohtaro and Isato, 2019). Several features of GA and its derivatives made them very attractive in commercial applications. GA is a versatile organic acid, which is noncorrosive, mildly acidic, less irritating, odorless, nontoxic, and easily degradable. Thus, as a food additive under the laws of some countries, GA and its derivatives are commonly added to dairy products, beverages and bakery to maintain flavor and prevent precipitation. For instance, taking advantage of the chelating ability of GA, Choi and Zhong added it to skim milk powder to reduce the turbidity of dispersions (Choi and Zhong, 2020).

This chelating ptoperty of GA also enable its numerous applications in the chemical fields. On one hand, GA can serve as a green solvent for metal extraction/coordination and organic synthesis. For example, GA is capable of recovering lithium and cobalt from related wasted materials (Roshanfar et al., 2018). On the other hand, it is a mild and eco-friendly metal cleaning agent for dishwashing, laundry, and water conditioning.

In the medicine industry, some divalent metal salts (Ca^2+^, Mg^2+^, Fe^2+^ and Zn^2+^) of GA can act as mineral nutrients to treat elemental deficiencies (such as osteoporosis and anemia) of humans and animals. Recent studies have shown that 1.25 mmol/L calcium gluconate (equivalent to the concentration of free calcium ions in mammalian blood) can disrupt the drug-resistant *Staphylococcus aureus* biofilm by hindering the formation of extracellular polymeric substance (EPS) matrix (Liu et al., 2021). Mycielska et al. proposed that gluconate can inhibit tumor growth and alter metabolic characteristics of tumor tissue via blocking citrate uptake (Mycielska et al., 2019). Moreover, GA and its derivatives can be overoxidized to synthesize keto-GA, glucuronic acid and glucaric acid, which are raw materials to produce some short-chain organic acids, such as formic acid, ascorbic acid, tartaric acid and xylonic (Stottmeister et al., 2005; De Muynck et al., 2007; Jin et al., 2016).

Chemical approaches and biological fermentation are mainly selected to produce GA in modern times. Although the one-step conversion to synthesize GA by chemical and electrolytic oxidation is efficient, the expensive electrolysis cost, environmental toxicity and biological hazards limit the industrial application of chemical approaches (Pal et al., 2016). With biological fermentation dominating industrial-scale production, industrial fermentation advances rapidly, making previous *Penicillium* spp. fermentation (Herrick and May 1928) replaced by the filamentous fungi fermentation and *Acetobacter* spp. submerged fermentation with a higher efficiency and specificity (Anastassiadis and Morgunov, 2007). Table 1 lists the reported microorganisms with application value for GA production in the last decades. The significant advantages of producing GA by fermentation stem from the use of various biomass materials as substrates and mature fermentation processes. In addition, GA can also be produced using immobilized enzymes (Singh et al., 2003; Singh, 2008) and immobilized cells (Blandino et al., 2001; Zhao et al., 2014) in fermentation. Nowadays, the cutting-edge research in the fermentation of GA focuses on the integrated routes, which mixes the use of agro-industrial byproducts, genetically engineered enzymes and more efficient bioreactor with purification systems.

**TABLE 1:** Gluconate-producing fungi and bacteria with application value in the last decades.

Gluconate-producing fungi	References	Gluconate-producing bacteria	References
***Aspergillus niger***	Ajala et al., 2017	*Acetobacter diazotrophicus*	Pal et al. (2016)
	Chuppa-Tostain et al., 2018		
	Ahmed et al. (2015)		
***Aspergillus carneus***	Lim and Dolzhenko, (2021)	*Acetobacter methanolicus*	Pal et al. (2016)
***Aspergillus terreus***	Ahmad Anas et al. (2012)	*Gluconobacter oxydans*	Pal et al., 2017
			Hou et al., 2018
			Yao et al., 2017
			Zhou and Xu, 2019
			Jiang et al. (2016)
***Aureobasidium pullulans***	Anastassiadis and Rehm, 2006a	*Gluconobacter japonicus*	Cañete-Rodríguez et al. (2016)
	Anastassiadis and Rehm, 2006b		
	Anastassiadis et al., 2003		
	Ma et al. (2018)		
***Penicillium variabile***	Crognale et al. (2008)	*Pseudomonas taetrolens*	Alonso et al. (2015)
***Penicillium puberulum***	Ahmed et al. (2015)	*Zymomonas mobilis*	Ferraz et al. (2001)
***Penicillium frequentans***	Ahmed et al. (2015)	*Azospirillum brasiliensis*	Rodriguez et al. (2004)
***Penicillium chrysogenum***	Ramachandran et al. (2006)	*Klebsiella pneumoniae*	Wang et al. (2016)
***Penicillium glaucum***	Ramachandran et al. (2006)	*Pseudomonas plecoglossicida*	Wang et al., 2018
***Penicillium notatum***	Ramachandran et al. (2006)	*Pseudomonas ovalis*	Pal et al. (2016)
***Penicillium oxalicum***	Han et al. (2018)	*Pseudomonas acidovorans*	Ramachandran et al. (2017)
***Saccharomyces cerevisiae***	Kapat et al. (2001)	*Pseudomonas fluorescens*	Sun et al. (2015)
		*Rhodotorula rubra*	Ramachandran et al. (2017)

For the past few decades, microbial fermentation routes have covered almost all production of GA and its derivatives with considerable progress. The properties, applications and sources of GA, as well as the typical microorganisms, fermentation methods and downstream processing during the fermentation are well summed up in a series of reviews (Anastassiadis and Morgunov, 2007; Singh and Kumar, 2007; Cañete-Rodríguez et al., 2016; Pal et al., 2016; Ramachandran et al., 2017). In this review, we first introduced the latest advancements in the fermentation of GA and its derivatives and focused on their regulatory mechanisms in fungi and bacteria. Then, the defects of these processes were discussed as well. Finally, we summarized the recent developments of cheap agro-industrial byproducts as carbon sources and the innovative integrated routes in the fermentation of GA and its derivatives.

## 2 Production of Gluconic Acid and Its Derivatives by Fermentation

In the global industrial fermentation market, organic acids fall into the third largest category of products, only next to antibiotics and amino acids. GA and its derivatives account for a large share in the organic acid market owing to their wide application (Anastassiadis and Morgunov, 2007). At present, the most commonly used strains in microbial fermentation to produce GA are *Aspergillus* spp. and *Gluconobacter* spp. Within these microbial factories, the industrial fermentation processes for GA production have been developed specifically for *Aspergillus niger and Gluconobacter oxydans*. Therefore, the GA synthesis mechanism and fermentation process of *A. niger* and *G. oxydans* are the most thoroughly studied.

### 2.1 Production Status and Recent Progress of Gluconic Acid and Its Derivatives

#### 2.1.1 Fungal Fermentation: *A. niger* as an Example

**FIGURE 2:**
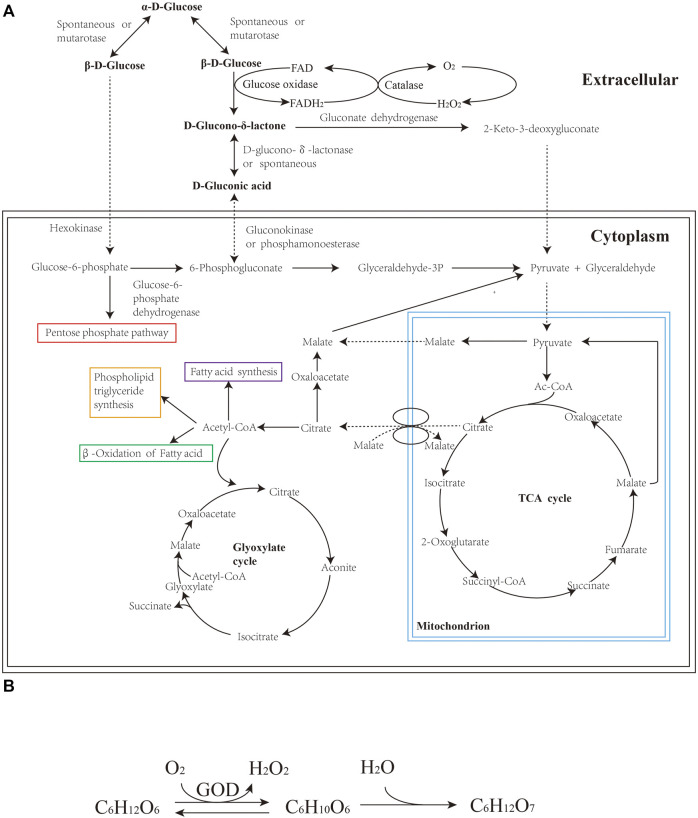
**(A)** Pathway of GA from glucose in fungi; **(B)** Overall reaction mechanism of GA in fungi.

*A. niger* is an ideal candidate for fermentation due to its robust growth, high yield and easy separation of GA products (Bankar et al., 2009). Currently, producing GA by industrial fermentation is mostly based on the specific fermentation process of *A. niger* which was patented by Blom in 1952 (Blom et al., 1952) and improved by Ziffer et al., in 1971 (Ziffer et al., 1971). In *A. niger,* glucose oxidase (GOD, EC 1.1.3.4) is the responsible enzyme for the catalytic oxidation of glucose to GA. GOD is a flavoprotein mainly present in cell walls and extracellular fluid, whose activity accounts for approximately 80% of the total enzymatic activity, as proved in both *Aspergillus* sp. and *Penicillium* sp. (Johnstone-Robertson et al., 2008; Bankar et al., 2009). The GOD-catalyzed oxidation reaction is an aerobic fermentation process with extremely high oxygen consumption. Figure 2A depicts the biochemical metabolism of GA production derived from *A. niger* involving the overall oxidation process. d-Glucose is converted into D-glucono-δ-lactone by a dehydrogenation reaction, during which H_2_O_2_ is generated as a byproduct. H_2_O_2_ is then decomposed into O_2_ and H_2_O in the presence of catalase. Figure 2B shows the overall reaction process from d-glucose to GA in fungi. D-glucono-δ-lactone undergoes spontaneous hydrolysis under neutral or alkaline conditions. As the reaction proceeds, the accumulation of GA makes the medium acidic, after which lactones are hydrolyzed by lactonase. Subsequently, GA penetrates cell walls and is then metabolized through the pentose phosphate pathway.

Submerged fermentation is commonly adopted in *A. niger* fermentation on an industrial scale. Sodium gluconate production accounts for more than 80% of the total production of GA derivatives in the globe (Roehr et al., 2001). Thus, sodium gluconate production by submerged fermentation of *A. niger* with glucose syrups (70 °Brix strength) is taken as an example here. Noteworthily, the seed medium for *A. niger* inoculation requires a low C/N ratio, while the fermentation medium needs a high C/N ratio in practice. For inoculum development, 10^6^ conidia cm^−3^ are inoculated into vegetative seed media to obtain pellet-like mycelia after incubation at 30°C for 15–24 h. Then the seed density of 20–50 pellets cm^−3^ of is selected for subsequent fermentation (Moresi and Parente., 2014). However, Lu et al. reported that the optimum seed morphology for GA biosynthesis was a dispersed pattern rather than pellets. The highest overall yield of 1.051 ± 0.012 g/g was achieved and the average GA production rate (21.0 ± 0.9 g/L^/^h) in the dispersed pattern was 39.1% higher than that in the case of small pellets (Lu et al., 2015).

During the fermentation process, a large amount of glucose (120–350 g/L) and nitrogen and phosphorus source at a low concentration (20 mM) should be contained in the fermentation medium, of which pH is kept at 4.5–6.5 by a neutralizer NaOH and the air flow should be maintained high (Cañete-Rodríguez et al., 2016). All fermentation factors are monitored under continuous automatic control, including ventilatory capacity, dissolved oxygen (DO), pressure, temperature, pH and foam level. The mycelia can be reused up to 5 times with the intermittent addition of glucose throughout the fed-batch cultivation. The conversion rate of glucose is quite high (about 9–15 g/L/h) with a yield of 0.97–1 g/g at pH of 6.0–6.5 around 34°C (Ramachandran et al., 2017). At present, batch fermentation is equipped with sensors to online monitor and control physicochemical parameters. On this basis, a calculated automatic feedback strategy was applied successfully in cell-recycle continuous fermentation with *A. niger*, which significantly increased the conversion rate of glucose (31.05 ± 0.29 g/L/h) with a yield of 0.984 ± 0.067 mol/mol (Lu et al., 2016a; Lu et al., 2016b).

The downstream purification of sodium gluconate from the fermentation medium relies on electrodialysis, anion/cation exchange and membrane separation. The mycelia are separated by using aseptic centrifugation or vacuum filtration from the cultivation fluid. Then the clarified cultivation fluid undergoes a series of steps such as filtration, precipitation, decolorization, neutralization, evaporation and crystallization to obtain sodium gluconate (98%) in the technical grade. Free GA, like 50% (w/w) GA aqueous solution, can be recovered by passing the concentrated sodium gluconate through a cation exchanger column to remove Na^+^ ions. Purified D-1,5-gluconolactone and D-1,4-gluconolactone can be obtained from supersaturated solutions of GA at 30–70°C and higher than 70°C respectively (Moresi and Parente., 2014). The conventional purification processes generate a huge amount of wastewater and demand relatively high manpower, leading to environmental pollution and expensive production cost. Thus, in the continuous batch-fermentation with a high cell density and GA concentration, the major technological bottleneck lies in product separation and purification from fermentation media. Several membrane integrated systems are combined with fermenters to improve the downstream process. Generally, microfiltration and ultrafiltration membranes are used to recycle the mycelia and proteins back to fermenters, Moreover, nanofiltration, reverse osmosis and electrodialysis membrane are adopted in separating GA product instead of the filtration of ion exchange in conventional downstream processes. Vikramachakravarthi et al. have used a neutral or charged nanofiltration membrane for the purification of GA and achieved a good result (Vikramachakravarthi et al., 2014).

#### 2.1.2 Bacterial Fermentation: *G. Oxydans* as an Example

**FIGURE 3:**
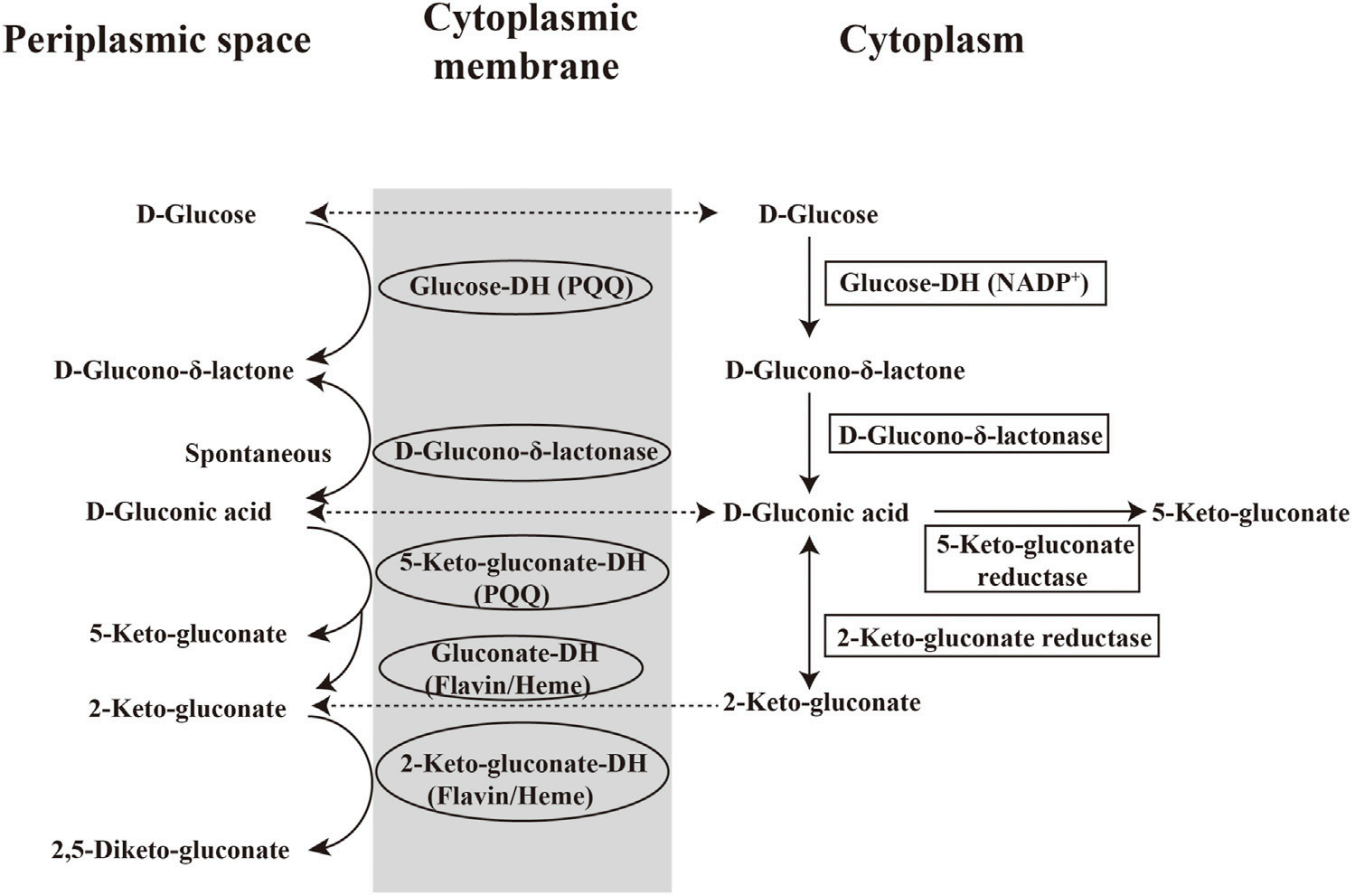
Pathway of GA from glucose in bacteria.

Different from GA production in fungi, the oxidation of glucose in bacteria is catalyzed by glucose dehydrogenase (GDH, EC 1.1.99.17). *G. oxydans* is preferred over *A. niger* in continuous fermentation because the chemostat cultivation cannot be performed on the latter. During the metabolism of *G. oxydans*, organics undergo incomplete oxidation (namely overflow metabolism) due to the lack of a glycolysis pathway and enzymes for tricarboxylic acid cycle (TCA). Therefore, *G. oxydans* oxidizes glucose to produce intermediate products like GA instead of CO_2_ and H_2_O. GA can be synthesized via two alternative pathways in *G. oxydans* (Figure 3). The first is the direct glucose oxidation pathway located in the periplasmic space and catalyzed by a membrane-bound PQQ-dependent GDH (EC 1.1.99.17). d-glucose is oxidized to D-δ-gluconolactone and then to D-GA which can be further oxidized to 2-keto-gluconate and 2,5-diketo-gluconate by flavin-dependent gluconate dehydrogenase and 2-keto-gluconate dehydrogenase, respectively. These two membranes-bound dehydrogenases can transfer electrons to the respiratory chain and produce the energy, while heme serves as the prosthetic group. Moreover, D-GA can also be oxidized to 5-keto-gluconate by a membrane-bound PQQ-dependent 5-keto-gluconate dehydrogenase. The second pathway is in the cytoplasm and the oxidation products flows to the pentose phosphate pathway at pH 7.5. In this pathway, d-glucose is transferred into cells and converted to D-GA by a NADP^+^-dependent glucose dehydrogenase (EC 1.1.1.47), then further oxidized to 2-keto-gluconate (at neutral pH) or 5-keto-gluconate (at acidic pH) by 2-keto-gluconate reductase and 5-keto-gluconate reductase, respectively. In either case, D-GA is mainly produced in the periplasm of *G. oxydans* where the activity of PQQ-dependent GDH is approximately 27-fold higher than that of NADP^+^-dependent GDH in cytoplasm (Pronk et al., 1989). The incomplete oxidation of glucose results in the accumulation of nearly quantitative amounts of the GA product outside the cells. GA is released into the medium via porins present in the outer membrane. Its genomic sequencing (Prust et al., 2005) reveal multiple metabolic pathways and confirms the synthetic pathways mentioned above.

*G. oxydans* is a strictly aerobic bacterium which can carry out highly effective continuous fermentation. It is also well known that the rapid growth of *G. oxydans* is difficult even in a complete medium due to its low ingestion of carbon sources. Maximum biomass of *G. oxydans* is achieved in the media containing D-mannitol or D-sorbitol as a carbon source. However, the oxidation reaction catalyzed by membrane-bound GDH is a nongrowth associated process (Macauley et al., 2001; Zhou et al., 2018). The key factor influencing the continuous fermentation of *G. oxydans* is improving glucose conversion rate while ensuring certain biomass. For this reason, the seed medium on which *G. oxydans* is inoculated usually contains D-mannitol (25 g/L) or D-sorbitol (50 g/L) and yeast extract (5 g/L) to obtain enough biomass, while the fermentation medium needs D-sorbitol (50 g/L) and quite high concentration of glucose for the balance of enough biomass and the higher yield of GA and derivatives (Banerjee et al., 2018; Zhou and Xu, 2019). In continuous culture, *G. oxydans* responds slowly to pH decrease, and lower pH (<4.5) is advantageous to the formation of the incomplete oxidation product because the pentose phosphate pathway is inhibited. Generally, the glucose oxidation catalyzed by membrane-bound GDH is dominant when the glucose concentrations is above 15 mM and pH below 3.5 (Ramachandran et al., 2017). Meanwhile, *G. oxydans* is highly sensitive to DO concentration, and the rate of oxygen transfer determines the production rate of GA from glucose during fermentations. Yuan et al. conducted a batch fermentation using *G. oxydans* for 5-keto-d-gluconate production, and obtained a maximum 5-keto-d-gluconate concentration of 117.75 g/L with a productivity of 2.10 g/L/h under the condition that DO was continuously maintained above 20% (Yuan et al., 2015).

Similar to fungal fermentation, downstream processing steps such as centrifugation, evaporation, product precipitation, liquid–liquid extraction and adsorption (with anion-exchange resin or activated carbon) are involved in removing the residual microbial cells, water and volatile compounds (Varnicic et al., 2015). Especially, the batch processes requiring frequent shut-down and start-up of a system lead to low productivity and high labor cost (Banerjee et al., 2018). Such limitations can be overcome via combining membrane systems with continuous fermentation to maintain a high cell density and the continuous removal of products (Pal et al., 2016).

### 2.2 Regulation of Synthesizing Gluconic Acid and Its Derivatives

#### 2.2.1 Fungal Regulation

GA production via fungal fermentation is directly related to GOD activity. Extensive experimental evidence shows that the activity of GOD can be markedly increased by the following factors: 1) the media containing glucose or the substrates whose hydrolysates are glucose; 2) increasing pH by adding an appropriate neutralizer (NaOH and CaCO_3_); 3) maintaining a high DO level in the fermentation broth; 4) keeping high cell membrane permeability and suitable osmotic potential by approaches such as adding surfactant-like matter to release periplasmic GOD. Mu et al. enhanced the thermostability and pH tolerance of GOD from *A. niger* by using in *silico* design which significantly improved the GA yield by more than twice, accompanied with almost complete conversion of 324 g/L d-glucose to GA within 18 h (Mu et al., 2019). Furthermore, with the increase in the fungal concentration, pH becomes an important parameter for GA production. It can produce many kinds of weak organic acids (such as citric acid, GA, and oxalic acid) whose accumulation depends on the medium pH during GA fermentation (Vassilev and Vassileva, 1992). pH lower than 3.5 can trigger the tricarboxylic acid cycle (TCA) and promote the formation of citric acid. The optimal pH for GA production by fungal fermentation is 4.5–6.0. For example, the optimum pH for *A. niger* fermentation is 5.5 (Znad et al., 2004).

Besides, GA synthesis is a strictly aerobic process sensitive to the DO content in the medium. It has been reported that the oxygen concentration gradient and the volumetric oxygen transfer coefficient are important monitoring indicators for fermentation, which can largely influence the transfer rate of oxygen from the gas phase to the liquid phase (Ramachandran et al., 2017). Tian et al. demonstrated that sufficient oxygen supply for improving sodium gluconate synthesis can not only directly affect the substrate but also regulates glucose metabolism to form sodium gluconate (Tian et al., 2018). Therefore, the air flow rate and the agitation rate are crucial to controlling the DO content in the medium. In most cases, the oxygen in fermenters is exactly the oxygen in the air, whereas some scholars introduce high-pressure pure oxygen into the medium for boosting the conversion efficiency. Shen et al. observed that the highest yield of sodium gluconate rose to 0.903 mol/mol in *A. niger* fermentation within 15.5 h when the inlet oxygen concentration was raised to 32% by regulating the appropriate proportions of air and pure oxygen to appropriate values, and the yield of main by-product (citric acid) also dramatically decreased from 1.36 to 0.34 g/L (Shen et al., 2017). Fernandes et al. reported that an increased oxygen transfer rate by enhancing air pressure up to 4 bar favored the GA production of by *Aureobasidium. pullulans* (Fernandes et al., 2021)*.*

H_2_O_2_, a byproduct of GA synthesis, plays an inhibitory role in GA production. Only the activity of reduced GOD is reported sensitive to H_2_O_2_ (Witteveen et al., 1992). Although there exists gluconolactonase in *A. niger* which can accelerate the conversion from glucono-δ-lactone to GA, some scholars recommend removing lactones from media because their accumulation is disadvantageous to both the rate of glucose oxidation and the production of gluconates (Roukas, 2000).

Mutagenizing strains is also needed by GA fermentation to improve the conversion efficiency of the substrate and reduce the cost of industrial production. So far, the maximal GA production of 76.3 g/L has been obtained from solid-state fermentation by *A. niger* ARNU-4, a mutant improved by genetic manipulation, which has a remarkable ability to utilize the untreated sugarcane molasses and tea waste as substrates (Sharma et al., 2007). A recombinant strain z19, which was constructed by simultaneously expressing the GOD and catalase from *A. niger* in the wild-type *Penicillium oxalicum* strain with cellulolytic ability and has been utilized for sodium gluconate production from cellulose (Han et al., 2018). Overexpressing the GOD gene made the transformant GOEX8 to produce considerably more calcium gluconate (160.5 ± 5.6 g/L) and higher GOD activity (1,438.6 ± 73.2 U/mg of protein) than its parent strain (Ma et al., 2018). The UV-induced mutant UV-112 of an acid-producing *A. niger* strain isolated from onions has a much higher glucose conversion rate than the original strain (0.66 g/g vs 0.25 g/g) (Prabu et al., 2012).

#### 2.2.2 Bacterial Regulation

The secondary oxidation of GA to keto-GA limits bacterial fermentation for the industrial production of GA. The formation of keto-GA can be inhibited when the glucose concentration is higher than 15 mM and the pH is lower than 3.5 (Sarkar et al., 2010). Under optimal industrial conditions, the GA production efficiency of *G. oxydans* is 75–80%, which is however significantly dependent on pH, glucose concentration, and air flow. For example, during the batch fermentation of *G. oxydans* for producing 5-keto-d-gluconate, DO needs to be continuously maintained above 20% to ensure the product concentration of 5-keto-d-gluconate to reach 117.75 g/L with a productivity of 2.10 g/L/h (Yuan et al., 2015). *G. oxydans* 621H (DSM 2343) is reported suitable for GA production via biological fermentation due to its high oxidizability even in the case of poor cell growth (Sievers and Swings, 2005). Different types of bacteria oxidize glucose in different pathways. *Acetobacter* spp. metabolize d-glucose through the pentose phosphate pathway and the TCA cycle, while *G. oxydans* can only metabolize glucose through the pentose phosphate pathway and the Entner-Doudoroff pathway due to its lack of a complete TCA cycle. In other bacteria with the ability to produce a high yield of GA (e.g., *Acidomonas methanolica* and *Gluconacetobacter diazotrophicus*), the oxidation of glucose to GA is assisted by PQQ-dependent GDH on the cytoplasmic membrane, along with synthesizing ATP in the electron transport chain (Krajewski et al., 2010).

Usually, bacteria can usually achieve the goal of increasing the production of GA and its derivatives after genetic modification. Wang et al. knocked out the gluconic acid dehydrogenase encoding gene of *Klebsiella pneumoniae*, which blocked the keto-GA pathway and increased GA production to 422 g/L (Wang et al., 2016). However, the pathogenicity of *K. pneumoniae* limits its industrial fermentation and commercial applications. The recombinant strain *G. oxydans* ZJU2 was constructed by knocking out a gluconate-2-dehydrogenase encoding gene and a pyruvate decarboxylase encoding gene and then expressing a exogenous GDH from *Xanthomonas campestris*. The recombinant strain was applied to batch fermentation to yield 5- keto-d-gluconate with quite high productivity of 2.10 g/L/h (Yuan et al., 2015). Zeng et al. knocked out the gluconate-5-dehydrogenase encoding gene in a competitive pathway and overexpressed the gluconate-2-dehydrogenase in the synthetic pathway of 2- keto-d-gluconate in *Gluconobacter japonicus*, which increased the titre of 2- keto-d-gluconate by 63.81% in shake flasks (Zeng et al., 2019). Table 2 summarizes the GA production by fermentation of high-yielding strains in recent years.

**TABLE 2:** Reported high-yield GA production in recent years.

Strains	Fermentation Type	GA (g/L)	Volumetric Productivity (g/L/h)	References
*A. niger*	batch culture in air-lift bioreactor	150	2.3	Klein et al. (2002)
*A. niger* AN151	submerged fermentation	330	21.0 ± 0.9	Lu et al. (2015)
*A. niger* JCM 5549	immobilization/batch	272	6.1	Matsui et al. (2013)
*A. niger* SIIM M276	batch	76.67	0.86	Zhang et al. (2016)
*A. niger* NCIM 548	immobilization/batch	92	2.04	Mukhopadhyay et al. (2004)
*A. niger* IAM 2094	batch	80–100	1.13	Ikeda et al. (2006)
*A. niger* ORS-4410	batch	80.60	0.131	Singh et al. (2003)
*A. niger* ORS-4410	semi-continuous batch	110.94	0.9375	Singh, (2008)
*A. pullulans* (De Bary) DSM 7085	continuous batch in a cascading operation of two bioreactors	350–370	12.7–13.9	Anastassiadis and Rehm, (2006b)
*P. variabile* P16	double feeding fed-batch	240	2.02	Crognale et al. (2008)
*G. oxydans* NBIMCC 1043	batch	148.5	9.03	Velizarov and Beschkov, (1994)
*G. oxydans* DSM2003	cascade hydrolysis/batch fermentation	118.9	1.65	Hou et al. (2018)
*Klebsiella pneumoniae Δgad*	fed-batch	422	4.22	Wang et al. (2016)

### 2.3 Bottlenecks Existing in Industrial Fermentation to Produce Gluconic Acid and Its Derivatives

Bacteria such as *G. oxydans* are more sensitive to high-concentration glucose during fermentation to produce GA and its derivatives. The secondary oxidation of GA is a double-edged sword for industrial production by fermentation. For one thing, it provides more possibilities for the fermentation of different keto-GA products. For another thing, a large amount of keto-GA is generated in this process which leads to difficult product separation of target products. Both the effective inhibition of various by-products in fermentation process and the separation and purification of the target products in downstream processes are the problems that need to be solved in the industrial bacterial fermentation routes to produce GA and its derivatives.

Fungal fermentation, such as *A. niger*, features a high yield and less byproduct produced, which is thus widely used to produce GA on an industrial scale. However, there are still many problems to be addressed in the process: 1) Filamentous fungi and CaCO_3_ cannot be reused in industrial fermentation. Biomass recycling is a common means in the fermentation industry for rational resource utilization and cost savings. However, filamentous fungi (e.g., *A. niger*) exhibit diverse forms during liquid fermentation, such as dispersed mycelia, clusters and balls (Tang et al., 2015). During industrial fermentation, mycelia are entangled with CaCO_3_, protein flocculants, and activated carbon, which hinders the secondary utilization of strains and CaCO_3_ due to difficult separation (Novalic et al., 1997). 2) The inoculated spores of *A. niger*-like mold for fermentation require to be cultured for a long time under complex culture conditions, and spore contamination is prone to occur (Anastassiadis S. and Rehm H. J., 2006). They can produce spore powder polluting the environment. Thus, workers are protected from spore powder-induced inhalation allergy through equipment and facilities in modern industrial fermentation. Mycotoxins can be produced from the metabolism of some *A. niger* species, and 3–10% of *A. niger* is positive in the detection of ochratoxin A and aflatoxin. This necessitates the toxin detection of new strains and those modified by molecular biological approaches or UV mutation under controllable fermentation conditions, especially for the food and medicine industries (Schuster et al., 2002). 3) *A. niger* also suffers from the failure to continuous fed-batch fermentation, a single carbon source type, and the curb of product synthesis by high-concentration carbon sources. Since the GOD in *A. niger* is highly active only in glucose-containing media, glucose or carbon sources with glucose as hydrolysates are used in the industrial fermentation of *A. niger*. Although *A. niger* shows a high conversion rate (>90%) in the case of proper glucose content, it can form excess mycelia during fermentation with high-concentration carbon sources, which diminishes the yield because of increasing the viscosity of media and reducing air flow and assimilation in medium (Ramachandran et al., 2017). Continuous fed-batch fermentation is a widely used method in the fermentation industry. However, it is infeasible to use glucose at a rather high concentration (>300 g/L) in *A. niger* fermentation (Roehr et al., 2001). The GOD in *A. pullulans* is less dependent on glucose, and economical carbon sources such as inulin can be utilized directly (Jiang et al., 2016). Anastassiadis and Rehm reported several methods to produce GA by continuous fermentation of *A. pullulans*, with the GA yield reaching 375 g/L and the product recovery 78% (Anastassiadis S. and Rehm H.-J., 2006). Thus, it has always been meaningful to develop new strains for industrial production of GA and its derivatives and combine the strains with suitable industrial production processes.

## 3 Agro-Industrial Byproducts as Substrates for the Productions of Gluconic Acid and Its Derivatives

Currently, many researchers are searching for new raw materials in hope of achieving high-added-value products with more economical and environmental-friendly methods (Wang et al., 2022). Hence, raw materials such as glucose and sucrose are gradually replaced by agro-industrial byproducts (e.g., hydrolysates of sugarcane, corn stover feedstock, corn cob, tea waste, starch, inulin, whey, figs, bananas, grapes, strawberry surpluses, wastepaper and lignocellulose) in the production of the fermentation for GA and its derivatives by fermentation. On one hand, these agro-industrial byproducts are rich in carbohydrates, the microbial fermentation of which enables the sustainable utilization of resources. Among numerous industrial and agricultural waste fermentation media, starch and polysaccharide media are superior to lignocellulose medium which requires pretreatment at high temperature, high pressure and low pH. Singh et al. obtained the mutant *A. niger* strain ORS-4.410 after continuous UV irradiation with excellent fermentability for saccharides derived from agricultural wastes (grapes and bananas) in batch fermentation and semi-continuous fermentation (Singh, 2008). Jordan et al. reported that the hydrolysis of lignocellulose can yield many compounds (e.g., carboxylic acids, furan aldehydes, and aromatic compounds) toxic to or suppressing microorganisms (Jordan et al., 2011). Zhang et al. also conducted tolerance tests on cell growth, GA production and GOD activity of *A. niger* SIIM M276 to corn stover hydrolysates (several furan derivatives, organic acids and phenolic compounds). They found that furfural influenced GA fermentation most, which can almost totally inhibit fungal metabolism at a concentration higher than 1.0 g/L (Zhang et al., 2014). Then researchers optimized the parameters of *A. niger* fermentation to produce GA and obtained 76.67 g/L GA in fermenters by using corn stover hydrolysates subjected to dry dilute acid pretreated (Zhang et al., 2016). However, the latest evidence showed that the acetic acid can act as an inhibitor for the oxidation from glucose to GA in *G. oxydans* fermentation to facilitate 2-keto-GA accumulation and improve gluconate dehydrogenase activity (Dai et al., 2022). Inspired by this, Zhou et al. co-produced xylooligosaccharides and GA from sugarcane bagasse with an economical approach involving the pre-hydrolysis treatment by acetic acid and obtained a GA yield of 96.3% by maintaining low pH stress (Zhou and Xu, 2019).

On the other hand, these diverse forms and different properties of agro-industrial byproducts as substrates also contribute to more changes in fermentation processes. Taking raw materials as substrates/supports has greatly promoted the development of the solid-state fermentation (SSF). Media, fermentation parameters and other factors including oxygen transfer, temperature, humidity, and aeration have been thoroughly studied. Sharma et al., evaluated the ability of SSF with tea waste as solid support to produce GA by *A. niger* (ARNU-4) strain, and optimized some parameters such as moisture content (70%), temperature (30 °C), aeration rate (2.5 L/min), and inoculum size (3%) for maximum GA production (76.3 g/L). Additives (e.g., jaggary, yeast extract, cheese whey, and mustard oil) with different concentrations were supplemented for further enhancing the production ability of *A. niger*. However, only yeast extract (0.5%) was proved an effective additive to enhance the GA production (82.2 g/L) (Sharma et al., 2007). Roukas et al. investigated the production of GA and citric acid with dry fig by SSF of *A. niger* ATCC 10577 and obtained the maximum transformation rate (685 g/kg) for GA from dry fig after adding 6% (w/w) methanol into the substrate (Roukas, 2000). To pursue a more efficient and economical fermentation process, Hou et al. carried out cascade hydrolysis and fermentation (CHF) without solid/liquid separation for GA and xylonic acids in a highly viscous hydrolysate slurry. The CHF process overcomes the inhibitiory effect of the intermediate glucono-1,4-lactone on cellulase activity and produced 118.9 g/L of GA and 59.3 g/L of xylonic acid (Hou et al., 2018).

## 4 Innovative Integrated Routes for the Production of Gluconic Acid and Its Derivatives

In moving toward a sustainable efficient technology regime nowadays, integrated production strategies are gradually adopted for the production of GA and its derivatives rather than conventional fermentation methods. Multiple biotechnologies have been combined in their production, such as immobilized cells, immobilized enzymes, enzymatic modification, polymer material, bimetallic catalysts, surface modification and novel reactors. Researchers have developed many alternative processes to minimize the production costs associated with raw materials and product separation. Ruales-Salcedo et al. proposed a hybrid process consisting of a multi-enzyme production system and *in situ* GA recovery by a liquid membrane in Taylor flow, which increased the GA productivity and yield to 1.6- and 1.7-fold higher than those in the multi-enzyme system containing sodium acetate buffer, respectively (Ruales-Salcedo et al., 2020). Yu et al. immobilized three types of enzymes (cellulase, GOD and catalase) on a reversible soluble Eudragit L-100 and then applied the system to the catalytic synthesis of GA from corn straw, with the conversion rate of cellulose to GA reaching 61.41% (Yu et al., 2021). Huang et al. also reported that ε-Poly-l-lysine was an ideal donor of amino to GOD and catalase. The immobilized GOD and catalase with surface amine modification were demonstrated 1.56 times higher activity recovery than that of before (Huang et al., 2018). DNA-guided enzyme assembly was reported that can enhance the production of GA directly from cellulose (Chen et al., 2017). Multiple studies have indicated that the integration of multi-stage micro- and nanofiltration in appropriate membrane units with various fermentation processes can significantly enhance the production efficiency of GA and its derivatives (Dekonda et al., 2014; Pal et al., 2016; Kuznetsov et al., 2017). Pal et al. obtained 93 g/L GA with a yield of 0.94 g/g by using the membrane-integrated hybrid fermentation system. The performance parameters of this system established the superiority of the novel technology over the traditional ones (Banerjee et al., 2018). Nevertheless, with the development of high-yielding strains for GA and the gradual maturity of industrial fermentation, integrated routes with more complicated operations and higher costs are more difficult to apply in industrial production. More concise and economical integrated production strategies are expected in the future.

## 5 Summary and Outlook

GA and its derivatives have broad application prospects in the food, medicine, construction and biotechnology industries. Microbial fermentation to produce GA has become a viable mode for commercial production. GA production is a simple one-step oxidative fermentation process. To reduce the cost of traditional fermentation, researchers have focused on the evaluation of cheaper agro-industrial byproducts for fermentation. This plays a significant role in promoting the development of GA fermentation in industrial practice. Meanwhile, they have also improved the fermentation process. Technological innovations have been merged recently with fermentation using agro-industrial byproducts to form the integrated routes. However, these integrated routes are rarely achieved in industrial fermentation applications. Some problems need to be tackled for greater benefits, including complicated reaction steps, fermentation waste discharge and a decrease in enzyme activity. Since the market demand is expanded increasingly as evident from foregoing reviews, current strains for industrial fermentation to yield GA face more and more challenges in production. Researchers are expected to keep screening and transforming strains with a strong ability for industrial production of GA, combining the advantages of fungal and bacterial fermentation while improving the fermentation process. Ultimately, all these efforts will pave the way for cheaper GA production by industrial fermentation, which would promote the combination of innovative GA production processes and industrial fermentation in practice.
